# Clinical characteristics, opportunistic infection spectrum and epidemiological analysis of pediatric AIDS patients in southwest China

**DOI:** 10.3389/fcimb.2026.1824149

**Published:** 2026-06-04

**Authors:** Yi Liao, Qi An, Chuan Wang, Dongmei Wang

**Affiliations:** 1 Department of Clinical Laboratory Medicine, Chengdu Women's and Children's Central Hospital, School of Medicine, University of Electronic Science and Technology of China, Chengdu, China; 2 Department of Science and Education Division, Public Health Clinical Center of Chengdu, Chengdu, Sichuan, China

**Keywords:** clinical characteristics, epidemiology, HIV/AIDS, opportunistic infection, pediatric

## Abstract

**Objectives:**

This study aimed to exploring the clinical, opportunistic infections, and epidemiological characteristics of hospitalized pediatric HIV/AIDS patients in southwest China.

**Methods:**

Retrospectively analyzed the clinical data of 87 hospitalized pediatric HIV/AIDS patients aged ≤14 years at the Public Health Clinical Center of Chengdu from January 2017 to September 2025, employing statistical methods to examine demographic characteristics, clinical indicators, infection spectrum, geographical distribution, and treatment outcomes.

**Results:**

The gender ratio of the affected children was 1.02:1, predominantly Han and Yi ethnic groups, concentrated in the minority regions of western Sichuan, with an average age of 8.70 ± 3.91 years. Parents of 48.3% of the children were AIDS patients, 55.2% received antiretroviral therapy (ART), and 70.0% had reduced CD4^+^T cell counts. Opportunistic infections were mainly candidiasis (45.3%) and tuberculosis (26.7%), with infection risk significantly negatively correlated with CD4^+^T cell counts, and gender-specific infection differences. The number of cases first increased and then decreased, with ART primarily using the AZT + 3TC+EFV regimen. 93.1% of the children improved and were discharged.

**Conclusion:**

Hospitalized pediatric HIV/AIDS patients in southwestern China is mainly transmitted from mother to child, with severe immune suppression at the time of diagnosis. The distribution of cases is related to medical resources. Although mother to child blockade and ART improve prognosis, there are still shortcomings in prevention and control in remote ethnic areas, and targeted screening, intervention, and medical resource allocation need to be strengthened.

## Introduction

Acquired Immune Deficiency Syndrome (AIDS) is a severe immunodeficiency infectious disease caused by Human Immunodeficiency Virus (HIV) infection. Children, due to their immature immune system, mother to child transmission, and faster progression of the disease, have become the most vulnerable and critical group in the prevention and treatment of HIV infection. The United Nations AIDS Programme estimates that 1.4 million children (aged 0-14 years) will be infected with HIV in 2024. HIV-infected children have far higher rates of opportunistic infections and mortality than adults, with longer treatment cycles and more complex interventions, further elevating pediatric HIV/AIDS public health costs and remaining a major global public health challenge (Patton et al., 2002; Pollock and Levison, 2023; Swinkels et al., 2025). In China, the HIV epidemic maintains a low overall prevalence but is extremely geographically imbalanced, with Sichuan Province in southwest China bearing a heavy epidemic burden (Cai et al., 2024; Li et al., 2024). In remote ethnic minority areas of the province, limited prenatal screening coverage, delayed intervention, poor treatment adherence, and severe immunosuppression at diagnosis lead to hospitalized HIV-infected children presenting with young age, severe immune deficiency, and complex complications, increasing treatment difficulty and further straining local public health and medical resources.

At present, there is still a relative lack of large-scale, systematic clinical and epidemiological research on HIV/AIDS among hospitalized children in Southwest China. Through retrospective analysis of the clinical data of children with HIV/AIDS hospitalized in the Public Health Clinical Center (PHCC), this study summarized the epidemiological characteristics, clinical manifestations and distribution characteristics of opportunistic infection, and discussed the key factors affecting the severity of the disease, aiming to provide evidence based basis for optimizing the early screening, standardized diagnosis and treatment, infection prevention and control, and health resource allocation of children with HIV/AIDS in the region, and help improve the comprehensive prevention and treatment of HIV/AIDS among children in southwest China.

## Methods

### Study design and population

**Figure 1:**
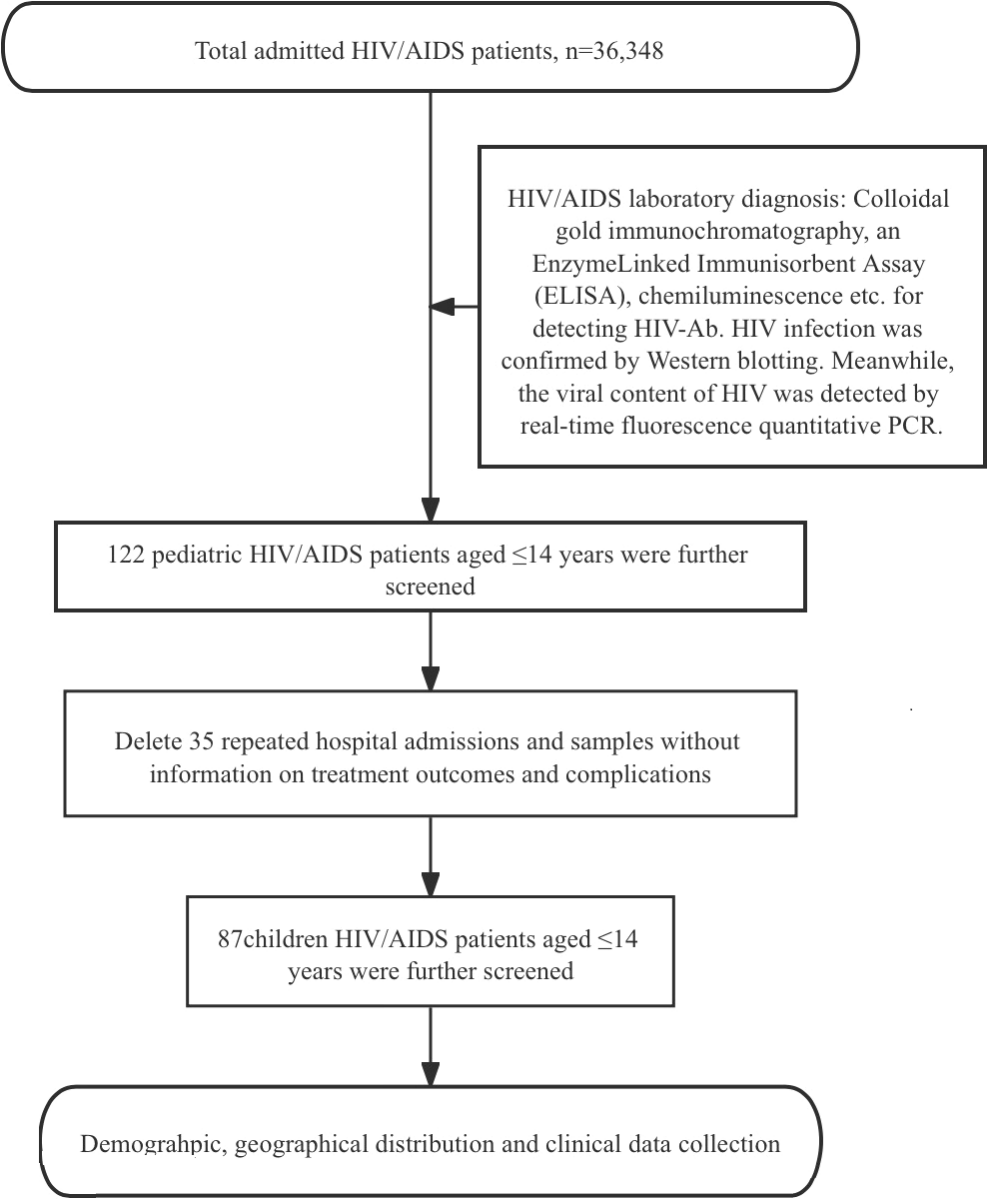
The flow diagram of our study. Demographic information and clinical data was reviewed from the Public Health Clinical Center of Chengdu, Sichuan, China; HIV, Human Immunodeficiency Virus; PCR, Polymerase Chain Reaction; AIDS, Acquired Immune Deficiency Syndrome.

This large-scale observational study was conducted in the PHCC, the AIDS quality control and the largest treatment center for HIV/AIDS in Sichuan Province, and is also a central unit for infectious disease management. Admitting about 2,500 HIV/AIDS patients for inpatient treatment every year. The present study included all HIV/AIDS patients admitted to the PHCC from January 2017 to September 2025. Based on etiology. HIV/AIDS cases were defined as colloidal gold immunochromatography, an EnzymeLinked Immunisorbent Assay (ELISA), chemiluminescence etc. for detecting HIV-Ab. HIV infection was confirmed by Western blotting. Meanwhile, the viral content of HIV was detected by real-time fluorescence quantitative polymerase chain reaction (PCR). Inclusion criteria (children aged ≤14 years with confirmed HIV/AIDS diagnosis) and exclusion criteria (repeated cases with multiple hospitalizations and cases with incomplete medical records). After excluding cases, a total of 87 pediatric HIV/AIDS patients aged ≤14 years were included in the study (**Figure 1**). Information from the hospital’s medical record management system and the information management system (HIS) was collected for each patient, including socio-demographic, clinical, therapeutic [e.g., use of antiretroviral therapy (ART)], geographic distribution of habitual residence, laboratory, and hospital outcome data etc. Information about the habitual residence was collected for all the pediatric HIV/AIDS cases, then coded according to their residence (geographic code), and matched to a 1:100000 digital map of China using Python 3.7.” The cases were displayed using a geographical distribution chart of China, Sichuan Province and Liangshan Yi Autonomous Prefecture.

### Diagnostic criteria

AIDS was diagnosed based on the Chinese AIDS and HIV infection diagnostic criteria (WS293–2008) (From the Centers for Disease Control and Prevention1993 revised classification system for HIV infection and expanded surveillance case definition for AIDS among adolescents and adults, 1993) In the laboratory of PHCC, the HIV detection methods we carry out include screening experiments: colloidal gold immunochromatography, an Enzyme-Linked Immunisorbent Assay (ELISA), chemiluminescence etc. for detecting HIV-Ab. HIV infection was confirmed by Western blotting. Meanwhile, the viral content of HIV was detected by real-time fluorescence quantitative PCR. The diagnosis of pediatric TB was based on the Chinese Pulmonary Tuberculosis Diagnostic Criteria (WS 288–2017), the Chinese TB Clinical Diagnosis and Treatment Guidelines (Chinese Medical Association, 2005), and the updated WHO guidelines (World Health Organization (WHO), 2014).

### Definitions of HIV-associated opportunistic infections

The diagnosis of opportunistic infections and AIDS-defining malignancies was conducted on the basis of the guidelines for prevention and treatment of opportunistic infections in HIV-infected children recommended by the Chinese guidelines for the diagnosis and treatment of human immunodeficiency virus infection/acquired immunodeficiency syndrome 2024 and United States Guidelines for the prevention and treatment of opportunistic infections in HIV-exposed and HIV-infected children (Siberry et al., 2013; Acquired Immunodeficiency Syndrome Professional Group, Society of Infectious Diseases, Chinese Medical Association and Chinese Center for Disease Control and Prevention, 2024). Opportunistic infections included pneumonia, tuberculosis, Talaromyces marneffei, candidiasis, hepatitis (B or C), syphilis, cytomegalovirus, herpesvirus, meningitis, and dermatitis. The diagnosis of pneumonia included bacterial pneumonia, viral pneumonia, pulmonary mycosis (including pneumocystis pneumonia), and pneumonia caused by other factors but did not include tuberculosis pneumonia, which was classified as tuberculosis (Jiang et al., 2019).

Diagnoses of HIV/AIDS and its opportunistic infections were also coinfected with medical history, clinical manifestations, X-ray imaging examinations, laboratory tests, and pathological data to perform comprehensive diagnosis. HIV-associated complications, including tumor, respiratory failure, septicemia, enteritis, hypoproteinemia and anemia, were diagnosed using the criteria in Internal Medicine.

### Ethical statement

This study was approved by the Ethics Committee of the PHCC (China) under the approval number YJ-K2023-08-01. As a retrospective study utilizing routinely collected patient data from the mandatory notification system, the Ethics Committee waived the requirement for informed consent.

### Statistical analysis

This study employed SPSS 26.0 software for statistical analysis. Count data were presented as frequency and percentage (%), with inter group comparisons conducted using Pearson χ² test. When theoretical frequency <5, Fisher’s exact test was applied. Multiple logistic regression model and ROC curve were used to analyze the key risk factors of hospitalized pediatric HIV/AIDS patients. A P value<0.05 was considered statistically significant.

## Results

### Demographic and clinical characteristics

This study included a total of 87 hospitalized pediatric HIV/AIDS patients, with 50.6% (44/87) being male and 49.4% (43/87) being female, resulting in a male to female ratio of 1.02:1. The average age was 8.70 ± 3.91, and the population distribution was mainly Han (41.4%) and Yi (40.2%) (χ ²=13.29, *P* = 0.010). The proportion of children receiving antiretroviral therapy was 55.2% (48/87), with the highest treatment rate in the 10-14 age group (65.4%) (χ ²=10.21, *P* = 0.006). The proportion of parents with HIV/AIDS accounted for 48.3% (42/87) The most common clinical symptoms are fever, anemia, cough, and expense. Anemia, bacterial pneumonia, and liver dysfunction are the most common complications related to pediatric HIV/AIDS. There was no statistically significant difference in the incidence of AIDS related complications among different age groups, including tumors, anemia, enteritis, sepsis, hypoalbuminemia, bacterial pneumonia, and mycoplasma pneumonia (all *P*>0.05) (**Table 1**). Among the pediatric AIDS patients, 70.0% had a decreased CD4 T-cell count (<200 cells/mL 56.3%, 200-349 cells/mL 20.7%). Laboratory results indicated that 37/42 (88.1%) had an elevated erythrocyte sedimentation rate (ESR), 33/60 (55.0%) and 16/55 (29.1%) had elevated levels of hypersensitive C-reactive protein and hyperprocalcitonin respectively. 28.7% (25/87) pediatric AIDS patients had leukopenia during treatment (**Table 2**).

**Table 1:** Demographic profile and clinical characteristics of pediatric HIV/AIDS patients in Southwest China, 2017–2025 (n = 87).

Variable	Total n=87 (% )	0-4 years n=15	5-9 years n=27	10-14 years n=45	χ 2	*P*	Variable	Total n=87 (% )	0-4 years n=15	5-9 years n=27	10-14 years n=45	χ 2	*P*
Sex							Dizzy	4 (4.6)	/	/	4 (8.9)	3.21	0.20
Male	44 (50.6)	5 (33.3)	15 (55.6)	24 (53.3)	2.19	0.5342	Shortness of breath	8 (9.2)	2 (13.3)	1 (3.7)	5 (11.1)	1.51	0.47
Female	43 (49.4)	10 (66.7)	12 (44.4)	21 (45.7)			Fatigue	8 (9.2)	/	3 (11.1)	5 (11.1)	3.01	0.22
Age group, Mean ± SD; years (range)	8.70 ± 3.91	15 (17.2)	27 (31.0)	45 (51.7)	0.00	0.9990	Vomit	5 (5.7)	/	2 (7.4)	3 (6.7)	1.11	0.57
Race/ethnicity							Consciousness disorder	1 (1.1)	/	1 (3.7)	/	1.01	0.60
Han nationality	36 (41.4)	7 (46.7)	11 (40.7)	18 (36.7)	13.29	0.0100	Palpitation	1 (1.1)	/	/	1 (2.2)	0.61	0.74
Tibetan	16 (18.4)	7 (46.7)	4 (14.8)	5 (10.0)			Hemoptysis	1 (1.1)	/	/	1 (2.2)	0.61	0.74
Yi	35 (40.2)	1 (6.7)	12 (44.4)	22 (43.1)			Myocardial injury	7 (8.0)	2 (13.3)	/	5 (11.1)	3.01	0.22
Receiving antiretroviral therapy	48 (55.2)	4 (26.7)	10 (37.0)	34 (65.4)	10.21	0.0060	Acute renal failure	1 (1.1)	/	/	1 (2.2)	0.61	0.74
Abnormal chest X-ray, n (%)	23 (26.4)	6 (40.0)	7 (25.9)	10 (18.9)	3.21	0.2010	Complication						
Patchy shadows or Nodules	20 (23.0)	6 (40.0)	6 (22.2)	8 (14.8)	3.01	0.2220	Tumor	3 (3.4)		1 (3.7)	2 (4.4)	0.21	0.897
Cavity	1 (1.1)	/	/	1 (1.8)			Anemia a	58 (66.7)	10 (66.7)	17 (63.0)	31 (68.9)	0.11	0.947
Hydropericardium	4 (4.6)	/	2 (7.4)	2 (3.6)	1.11	0.5740	mild anemia	39 (44.8)	6 (40.0)	11 (40.7)	22 (48.9)	0.61	0.738
Length of hospitalization (days), median (IQR)							moderate anaemia	16 (18.4)	3 (20.0)	5 (18.5)	8 (17.8)	0.20	0.905
<7	26 (29.9)	10 (66.7)	6 (22.2)	10 (18.9)	14.21	0.0070	severe anemia	3 (3.4)	1 (6.7)	1(3.7)	1 (2.2)	0.81	0.666
7-14	28 (32.2)	3 (20.0)	10 (37.0)	15 (25.4)			Enteritis	9 (10.3)	1 (6.7)	4 (14.8)	4 (8.9)	1.31	0.519
>14	33 (37.9)	2 (13.3)	11 (40.7)	20 (33.3)			Sepsis	7 (8.0)	3 (3.4)	3 (3.4)	1 (1.1)	4.21	0.122
One or both parents to AIDS	42 (48.3)	9 (60.0)	14 (51.9)	19 (31.1)	6.21	0.0450	Hypoproteinemia	13 (14.9)	4 (26.7)	3 (11.1)	6 (13.3)	1.11	0.574
Clinical characteristics							Bacterial pneumonia	45 (51.7)	7 (46.7)	13 (48.1)	25 (55.6)	0.10	0.951
Headache	4 (4.6)	/	1 (3.7)	3 (4.8)	1.0100	0.6030	Mycoplasma pneumoniae,Mp	5 (5.7)	1 (6.7)	2 (7.4)	2 (4.4)	0.21	0.897
Fever	44 (50.6)	12 (80.0)	15 (55.6)	17 (26.6)	12.31	0.0020	Liver dysfunction b	33 (37.9)	9 (60.0)	2 (7.4)	22 (48.9)	18.31	<0.001
Night sweat	1 (1.1)	/	1 (3.7)	/	2.11	0.3480							
Diarrhea	7 (8.0)	2 (13.3)	1 (3.7)	4 (6.1)	1.21	0.5460	Disease outcome						
Cough and expectoration	60 (69.0)	12 (80.0)	17 (63.0)	31 (68.9)	2.21	0.3320	Get better	81 (93.1)	14 (93.3)	25 (92.6)	42 (93.3)	4.11	0.39
Emaciation	23 (26.4)	6 (40.0)	6 (22.2)	11 (24.4)	2.31	0.3150	Othersc	5 (5.7)	/	2 (7.4)	3 (6.7)		
Rash	7 (8.0)	2 (13.3)	1 (3.7)	4 (8.9)	1.21	0.5460	Death	1 (1.1)	1 (6.7)	/	/		

a Anemia, mild anemia, hemoglobin 90 g /L – normal lower limit; Moderate anaemia, hemoglobin 60-89 g/L; Severe anemia, hemoglobin 30-59 g/L; Hemoglobin normal lower limit, 0.5–4.99 yrs. Hemoglobin < 110 g /L, 5–11.99 yrs. Hemoglobin < 115 g/L, 12–14.99 yrs. Hemoglobin < 120 g/L [6]; b Liver dysfunction is defined as various factors such as alcohol consumption, medication, and viruses can lead to acute or chronic liver function decline, with liver enzymes (Alanine Aminotransferase and Aspartate Aminotransferase) exceeding the normal value by 1.5 times or more;Myocardial injury refers to the clinical diagnosis of myocardial enzyme testing, electrocardiogram, cardiac ultrasound, etc;c Others:Transferred to another hospital or requested early discharge for personal reasons.

**Table 2:** Laboratory findings of pediatric HIV/AIDS patients in southwest of China (n = 87).

Variable	Total n=87 (% )	0-4 years n=15	5-9 years n=27	10-14 years n=45	χ ^2^	*P*
High precision HIV virus RNA (copies/mL), median (IQR)	1.39E+06	2.56E+06	1.06E+06	1.42E+06		
White blood cell counts <3.5*10^9^/L	25 (28.7)	3 (20.0)	15 (55.6)	7 (15.6)	11.21	0.004
Platelet (<100 ×10^9/L)	16 (18.4)	3 (20.0)	3 (11.1)	10 (22.2)	1.31	0.519
CD4+T cell counts when admitted to hospitl (cells/mL)						
<200	49 (56.3)	9 (60.0)	15 (55.6)	25 (55.6)	0.31	0.989
200-349	18 (20.7)	3 (20.0)	6 (22.2)	9 (20.0)		
≥350	20 (23.0)	4 (26.7)	5 (18.5)	11 (24.4)		
ESR (Female > 20, male > 15 mm / hour)	37/42 (88.1)	6/7 (85.7)	19/21 (90.5)	12/14 (85.7)	1.21	0.546
Super C-reactive protein (> 6 mg / L)	33/60 (55.0)	8/11 (72.7)	12/18 (66.7)	13/31 (41.9)	6.21	0.045
PCT (> 0.5ng/ml)	16/55 (29.1)	6 (40.0)	5/15 (33.3)	5/25 (20.0)	4.21	0.122

### The distribution of opportunistic infection spectrum in pediatric AIDS patients

**Figure 2:**
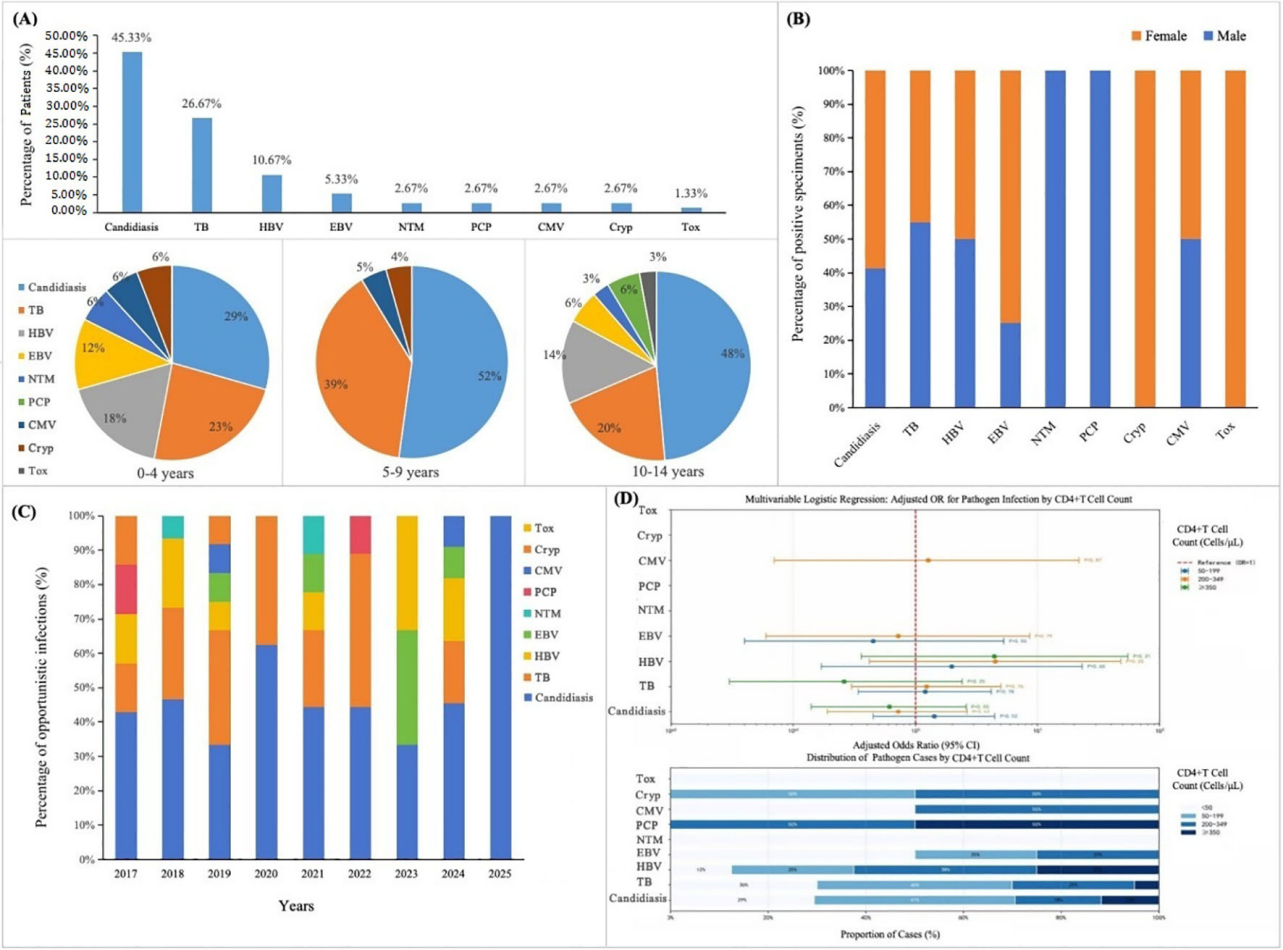
Opportunistic infection spectrum of hospitalized pediatric HIV/AIDS patients from 2017 to 2025 at Public Health Clinical Center of Chengdu, Sichuan, China. **(A)** Distribution of opportunistic infection pathogens, stratified by age. **(B)** Gender distributions of opportunistic infection pathogens. **(C)** Yearly distributions of opportunistic infection pathogens. **(D)** Distribution of opportunistic infection pathogens, stratified by CD4+T lymphocyte count. TB, tuberculosis; HBV, hepatitis B virus; EBV, Epstein-Barr virus; NTM, non-tuberculous mycobacteria; PCP, pneumocytsis carinii pneumonia; CMV, Cytomegalovirus; Crypj, cryptococcosis; Tox, toxoplasma gondii.

**Figure 3:**
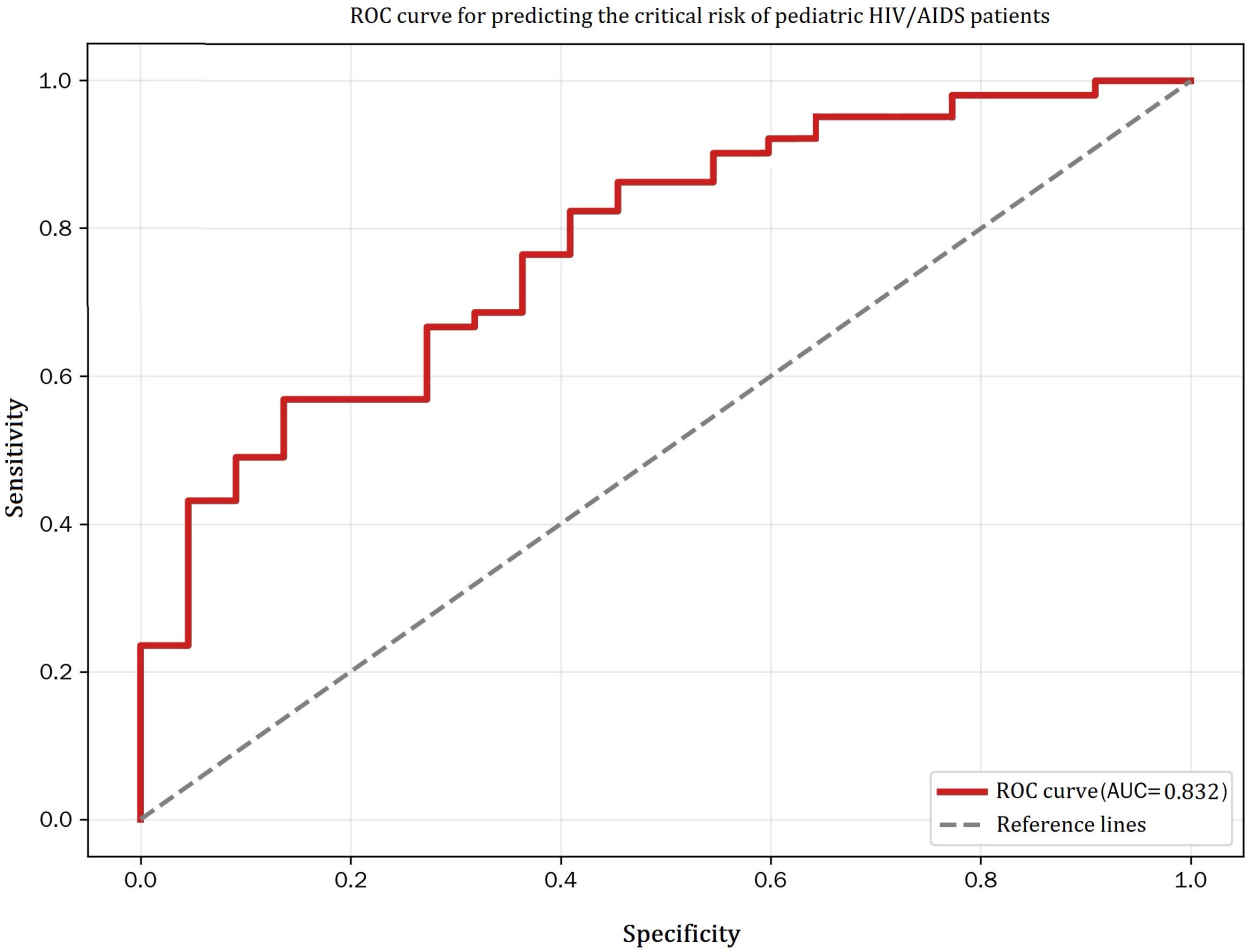
ROC curve for predicting the critical risk of pediatric HIV/AIDS patients.

Among the 87 hospitalized pediatric AIDS patients, candidiasis (45.3%), tuberculosis (26.7%), and HBV (10.7%) were the most common opportunistic infections. Boys are more susceptible to antibody (NTM) and pneumocytsis carinii pneumonia (PCP) infections, while girls are more susceptible to cryptococcosis and toxoplasma gondii (Tox). The risk of opportunistic infections in pediatric HIV/AIDS patients is significantly negatively correlated with CD4+T cell counts, tuberculosis and candidiasis in the high CD4 group (≥350), the adjusted OR< 1, indicating a significantly reduced risk of infection when immune function is normal (**Figure 2D**). Multivariate logistic regression showed that length of hospital stay (OR = 1.09, 95%CI: 1.03–1.16, *P* < 0.05), AIDS−related opportunistic infection (OR = 2.77, 95%CI: 1.45–5.28, *P* < 0.01), tuberculosis (OR = 2.29, 95%CI: 1.24–4.21, *P* < 0.05), and candidiasis (OR = 2.09, 95%CI: 1.15–3.80, *P* < 0.05) were independent risk factors for critical illness. CD4 category (OR = 0.42, 95%CI: 0.27–0.65, *P* < 0.01) and ART (OR = 0.51, 95%CI: 0.28–0.92, *P* < 0.05) were protective factors. The model had good performance with AUC = 0.832 (95%CI: 0.78–0.94, *P* < 0.001) (**Table 3**, **Figure 3**). There was no significant time trend in the opportunistic infection spectrum of pediatric HIV/AIDS patients between 2017 and 2025, and there was no statistically significant difference in the distribution of opportunistic infection spectra among different age groups (**Figure 2**).

**Table 3:** Multivariate logistic regression analysis of critical illness related factors.

Variable	OR	95% CI	*P* value
Gender	1.2	0.68–2.12	0.52
Age	1.06	0.97–1.16	0.18
Length of hospital stay	1.09	1.03–1.16	**<0.05**
CD4 category	0.42	0.27–0.65	**<0.01**
Parental HIV infection	1.23	0.71–2.13	0.45
ART	0.51	0.28–0.92	**<0.05**
AIDS-related OI*	2.77	1.45–5.28	**<0.01**
TB	2.29	1.24–4.21	**<0.05**
Candidiasis	2.09	1.15–3.80	**<0.05**

ART, antiretroviral therapy; OI, Opportunistic infection;TB, tuberculosis.The bold values P value<0.05/0.01 was considered statistically significant.

### Disease incidence trend and geographical distribution

**Figure 4:**
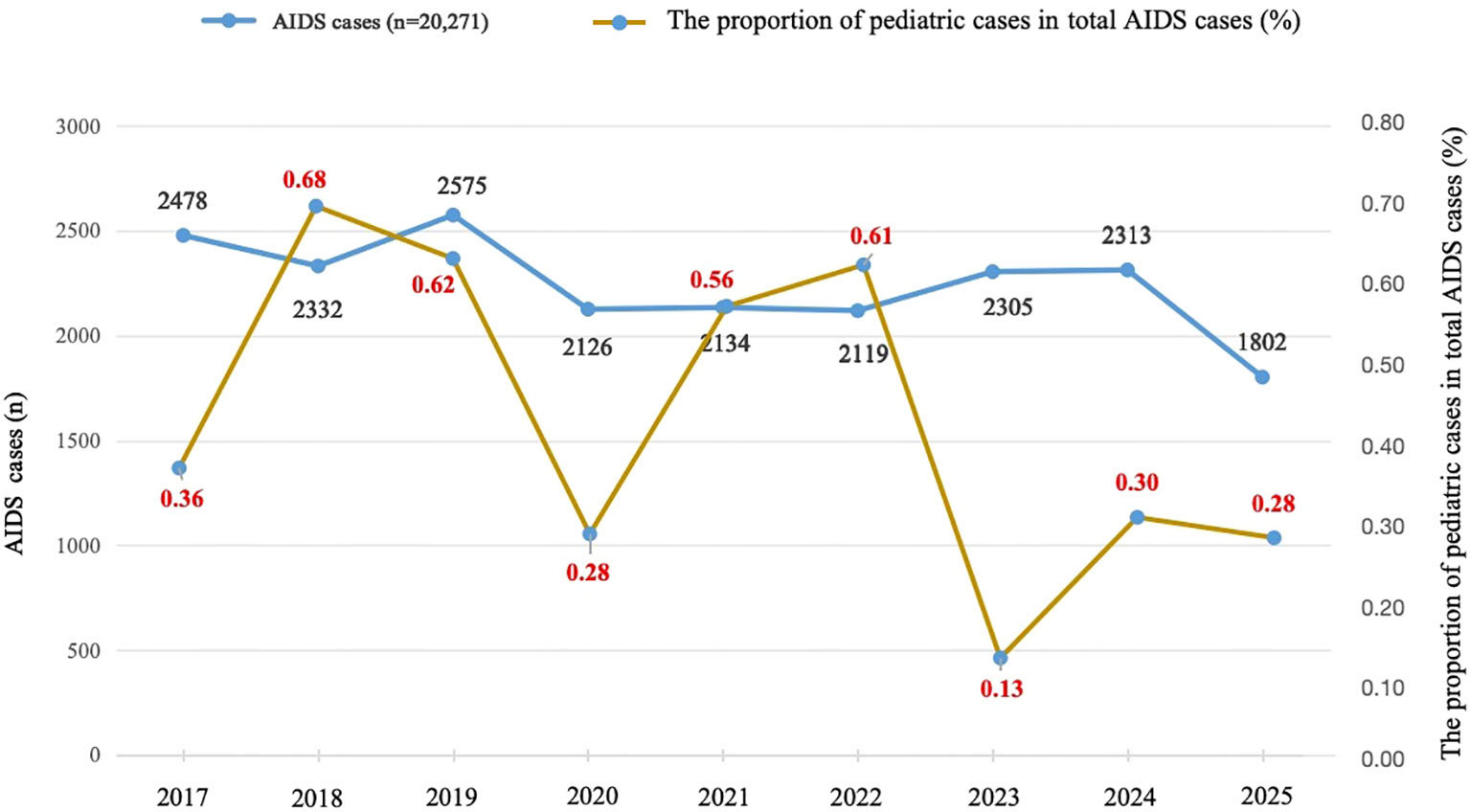
Numbers of hospitalized HIV/AIDS patients presenting each year between 2017 and 2025 (n =20, 271).

**Figure 5:**
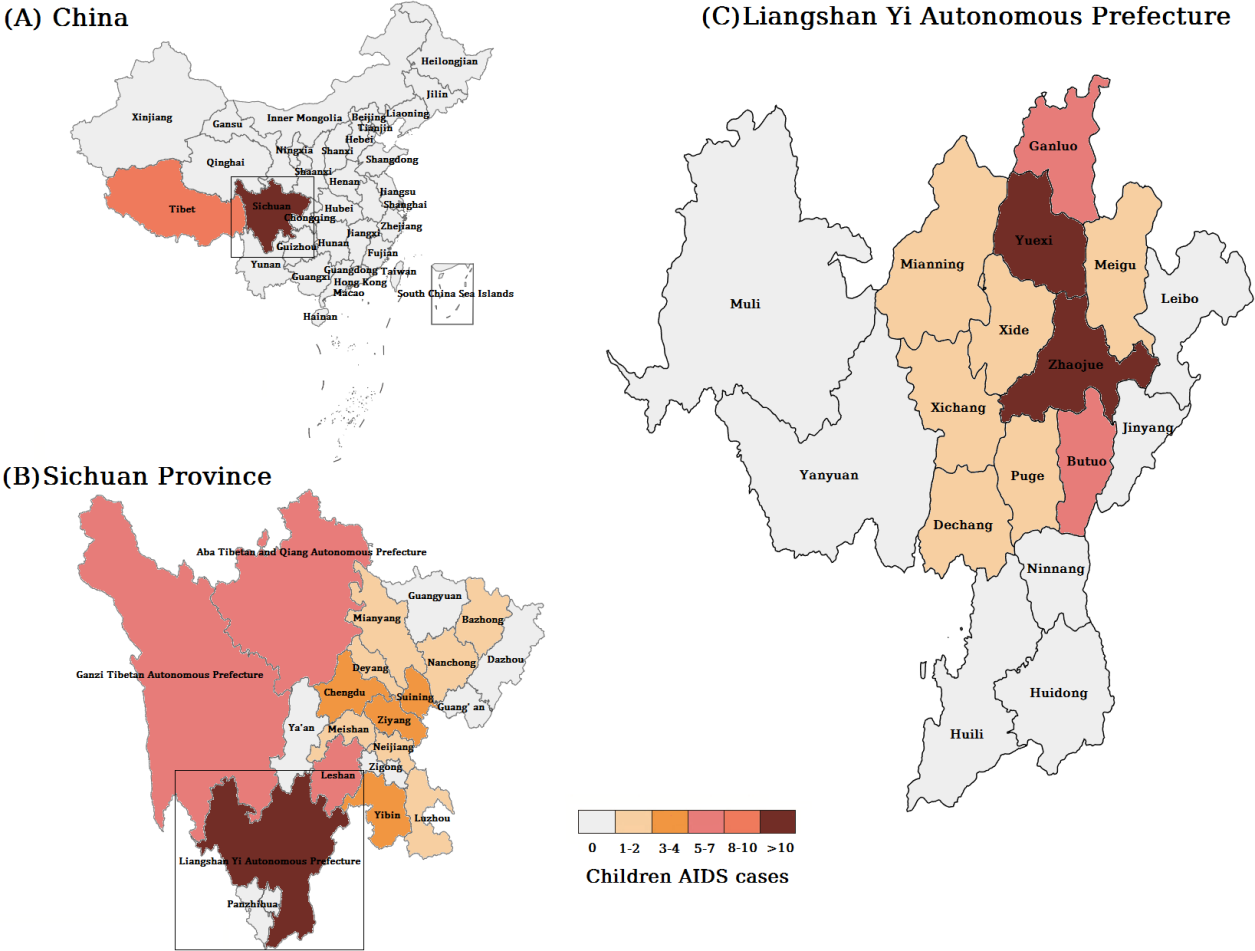
Geographical distribution of hospitalized pediatric HIV/AIDS cases in our study. **(A)** The geographical distribution of pediatric HIV/AIDS cases in southwest China. **(B)** The geographical distribution of pediatric HIV/AIDS cases in Sichuan. **(C)** The geographical distribution of pediatric HIV/AIDS cases in Liangshan Yi Autonomous Prefecture.

Among 87 pediatric HIV/AIDS patients from 2017 to 2025 included in this study, number of cases showed a trend of first increasing and then fluctuating downward. The overall number of AIDS cases reached its peak in 2019 (2575 cases), remained relatively stable at 2119-2134 cases from 2020 to 2022, and slightly rebounded from 2023 to 2024. Pediatric HIV/AIDS patients accounted for 0.43% of the total cases, Over the past 9 years, the proportion of pediatric cases in the total AIDS cases has shown two peaks in 2018 (0.68) and 2022(0.61), decreasing in 2020 (0.28), and reaching its lowest point in 2023 (0.13) (**Figure 4**). The geographical distribution map indicated that over the 9-year period, four of the cases were from neighboring cities Tibet outside Sichuan Province, while the remaining cases were from within Sichuan Province, primarily from the western Sichuan, Liangshan, Ganzi, and Aba minority areas, with the highest number of cases in the Liangshan Yi Autonomous Prefecture. The remaining cases were mainly distributed in Leshan, Chengdu, Ziyang and other central urban areas. The geographical location map of Liangshan Yi Autonomous Prefecture further shows that pediatric HIV/AIDS cases are mainly distributed in Yuexi, Zhaojue, Butuo, and Ganluo areas (**Figure 5**).

### Treatment and outcomes

Among the 87 pediatric HIV/AIDS patients, 48 cases had received or were receiving ART. Due to delayed diagnosis, insufficient family awareness, and the difficulty in managing young children, 39 cases did not receive ART. The treatment plan with the highest frequency of use was, zidovudine (AZT)+lamivudine(3TC)+efavirenz (EFV) (10 cases), followed by abacavir (ABC)+3TC+ritonavir-boosted lopinavir (LPV/r) (7 cases), ABC + 3TC+dolutegravir (DTG) (4 cases), AZT + 3TC+(LPV/r) (4 cases), AZT + 3TC+Nevirapine (NVP) (4 cases), ABC + 3TC+EFV (3 cases). Some cases may suspend medication or adjust the treatment plan during the medication period due to allergy or drug resistance. Among the 87 pediatric AIDS patients, 81 were discharged after their condition improved and was controlled, while 5 patients requested early discharge for personal reasons. One case of severe pneumonia and respiratory failure resulted in unsuccessful rescue efforts and death.

## Discussion

Through retrospective analysis of the clinical data of 87 hospitalized children with HIV/AIDS in PHCC, Sichuan Province, southwest China from January 2017 to September 2025.This is one of the pediatric cohorts with the longest hospital stay in China, systematically elaborated the epidemiological characteristics, clinical characteristics, distribution of opportunistic infection spectrum and diagnosis and treatment outcomes of this group in southwest China, providing a large sample of evidence-based data for the comprehensive prevention and treatment of AIDS among children in the region. The research results are highly consistent with the regional medical status and demographic characteristics of southwest China, and also highlight the focus and difficulty of the prevention and treatment of AIDS among children in the region.

The male to female ratio of HIV/AIDS patients included in this study is close to 1:1, with no significant gender difference, which is consistent with the gender distribution characteristics of HIV infection in children in some regions of China (Zhao et al., 2006; Wang et al., 2021). This suggests that gender factors have no significant impact on HIV infection in children with mother to child transmission as the main route. The ethnic composition of children is mainly Han and Yi, accounting for more than 80%, and the cases are highly concentrated in Liangshan, Ganzi, Aba and other minority areas in western Sichuan, which is highly related to the regional differences in the AIDS epidemic in southwest China (Yang et al., 2023).

There are problems such as frequent population mobility, insufficient coverage of mother infant screening, and relatively scarce medical resources in ethnic minority areas in western Sichuan, which have led to delayed HIV testing and intervention during pregnancy, making it a high-risk area for HIV infection in children. However, cases in central cities such as Chengdu and Leshan are mostly sporadic, reflecting that the accessibility of medical resources is a key factor affecting the prevention and control of HIV infection in children. The average age of the affected children is 8.70 ± 3.91 years old. The proportion of antiretroviral therapy (ART) in the 10-14 age group is the highest, reaching 65.4%, while the proportion of treatment in the younger group is relatively low. On the one hand, the clinical symptoms of younger children are hidden, and the diagnosis time is later. On the other hand, it is also related to poor medication compliance and insufficient treatment awareness among family members. The proportion of parents with one or both AIDS patients is 48.3%, which directly confirms that mother to child transmission is the core pathway of HIV infection in children in this region (Zhao et al., 2006; Yang et al., 2017), and also suggests that prevention and control education and intervention at the family level urgently need to be strengthened.

In this study, 70.0% of the children had a decrease in CD4+T cell count, with over half of the children having CD4+T cell count<200 cells/mL, accompanied by significant increases in inflammatory indicators such as erythrocyte sedimentation rate and high-sensitivity C-reactive protein. This suggests that hospitalized children with HIV/AIDS in southwestern China are mostly in a state of severe immune suppression when diagnosed, which is directly related to the lack of early screening and delayed diagnosis in remote areas, and is also the core pathological basis for the high incidence of opportunistic infections in this group. The most common clinical symptoms of children with HIV/AIDS are fever, weight loss, cough, and sputum. Complications mainly include anemia, bacterial pneumonia, and liver dysfunction, and there is no significant difference in the incidence of complications among different age groups, indicating that systemic inflammation and organ damage caused by immune deficiency are common clinical features of HIV/AIDS in children (PENTA Steering Committee, 2011; Zhao et al., 2013). The incidence of leukopenia is 28.7%, which also reflects the significant impact of HIV infection and treatment on children’s hematopoietic function. In clinical diagnosis and treatment, attention should be paid to hematopoietic function protection and symptomatic supportive treatment.

Opportunistic infection is the primary cause of the progress of HIV/AIDS in children. This study shows that candidiasis (45.3%), tuberculosis (26.7%), and hepatitis B virus infection (10.7%) are the most common opportunistic infections in this region, with a constituent ratio significantly higher than other infection types, and there is a significant gender difference. Boys are prone to be associated with nontuberculous mycobacteria (NTM) and pneumocystis pneumonia (PCP), and girls are more prone to be infected with cryptococcus and toxoplasma gondii. This gender difference may be related to different immune response characteristics and exposure environments of boys and girls, so it is necessary to carry out targeted screening in clinical practice. Of particular importance is that the risk of opportunistic infections is significantly negatively correlated with CD4 ^+^ T cell counts. Trend tests for tuberculosis, candidiasis, and total infections all suggest that the lower the immune function, the higher the risk of opportunistic infections. This is consistent with research findings on HIV/AIDS in children both domestically and internationally (Dankner et al., 2001; Zhao et al., 2013; Gebrerufael, 2023). It has been confirmed that early initiation of ART and reconstruction of immune function are key factors in preventing opportunistic infections (Zhao et al., 2013). There was no significant time trend in the opportunistic infection spectrum of this group from 2017 to 2025, and there was no statistical difference in the distribution of infection spectrum among different age groups, suggesting that the types of opportunistic HIV/AIDS infection among children in southwest China were relatively stable, and candidiasis and tuberculosis were always the focus of prevention and control, which was related to the epidemic background of fungi and tuberculosis in the region (Wang et al., 2025), and also reflected that preventive interventions against such opportunistic infections still needed to be strengthened.

In this study, the number of pediatric HIV/AIDS cases showed a trend of first increasing and then fluctuating downward. After reaching its peak in 2019, it gradually declined and reached its lowest point in 2023. Moreover, the proportion of pediatric cases to the total number of HIV/AIDS cases was only 0.43%. PHCC, as a designated AIDS quality control center in Southwest Sichuan Province, China, has a certain representativeness in the cases it treats. Therefore this trend to some extent reflects the effectiveness of China’s maternal and child prevention strategy in the southwest region. With the full coverage of HIV screening during pregnancy and timely initiation of maternal and child prevention interventions, new HIV infections in children have been effectively controlled. However, cases are still highly concentrated in ethnic minority areas in western Sichuan, with counties such as Yuexi, Zhaojue, Butuo, and Ganluo in Liangshan Yi Autonomous Prefecture being the core distribution areas of cases.

This suggests that the problem of uneven distribution of regional medical resources has not been fundamentally solved, and the prevention and control of mother and baby blockage, early screening, follow-up management, and other work in remote ethnic areas are still shortcomings. In terms of diagnosis and treatment outcomes, 93.1% of the 87 patients improved after treatment and were discharged from the hospital. Only 1 patient died of severe pneumonia or respiratory failure despite rescue efforts. The overall prognosis was good, and the ART regimen mainly consisted of zidovudine (AZT), lamivudine (3TC), and efavirin (EFV). Although this regimen has good tolerance and is easy to administer, making it suitable for clinical use in children (Consolidated guidelines on HIV prevention, testing, treatment, service delivery and monitoring: Recommendations for a public health approach, 2021). The latest guidelines and studies recommend the use of a treatment regimen based on DTG (Panel on Antiretroviral Therapy and Medical Management of Children Living with HIV, 2025; White et al., 2025). This may be due to the historical cohort effect during the 9-year inclusion period in this study, as well as the difficulty in obtaining new drugs for most cases in remote areas, resulting in slightly fewer DTG based treatment options in this retrospective study. In addition, some children may temporarily suspend or adjust their treatment plans due to allergies or drug resistance, indicating the need to strengthen monitoring of adverse drug reactions and drug resistance testing in ART treatment for children, and optimize treatment plans in a timely manner. At the same time, 5 patients were discharged early due to personal reasons, reflecting that family treatment compliance is still an important factor affecting treatment effectiveness, and it is necessary to strengthen doctor-patient communication and health education.

This study also has certain limitations: as a single center retrospective study, the included cases were all hospitalized patients, the findings primarily reflect the clinical burden and disease spectrum among tertiary-care inpatients, which cannot fully reflect the prevalence rate at the full regional pediatric HIV/AID landscape; Some patients’ medical records have missing indicators, which may have a certain impact on the statistical results; Without long-term follow-up of the affected children, it is impossible to analyze the long-term efficacy and prognostic factors of ART treatment. Subsequent research can conduct multicenter, prospective cohort studies, including outpatient and inpatient children, expanding the sample size, and increasing long-term follow-up links to analyze the effects of different ART regimens and intervention timing on immune function reconstruction, opportunistic infection prevention, and long-term survival of HIV/AIDS patients in children.

## Conclusions

Pediatric HIV/AIDS in southwest China is mainly transmitted through mother-to-child routes, mostly concentrated in ethnic minority areas of western Sichuan. Medical resources are insufficient, and screening and treatment compliance need to be improved. In the future, it is necessary to strengthen mother-to-child blocking, early screening and standardized treatment, improve follow-up management, and reduce the burden of pediatric infection.

## Data Availability

The original contributions presented in the study are included in the article/supplementary material. Further inquiries can be directed to the corresponding author.
